# Recent Progress on Affibody-Based Supramolecular Architectures: Moving from Monomeric Constructs to Multivalent Assemblies

**DOI:** 10.3390/ph18111669

**Published:** 2025-11-04

**Authors:** Hongfei Wang, Liqiang Wei, Chunyue Du, Antony Kam, Shining Loo

**Affiliations:** 1 Wisdom Lake Academy of Pharmacy, Xi’an Jiaotong-Liverpool University, Wuzhong No. 111, Renai Road, Suzhou 215123, China; 2 Department of Biosciences and Bioinformatics, School of Science, Xi’an Jiaotong-Liverpool University, Wuzhong No. 111, Renai Road, Suzhou 215123, China

**Keywords:** affibody, supramolecular architecture, therapeutics, diagnostics, drug delivery

## Abstract

Affibody molecules have emerged as versatile protein engineering platforms due to their exceptional binding properties. These small (6.5 kDa) three-helix bundle proteins, derived from the Z-domain of Staphylococcal protein A, can be engineered to bind diverse molecular targets with high affinity and specificity. This structural and functional versatility has driven their applications in diagnostics, therapeutics, and biosensing. This review examines the evolution from monomeric affibody constructs to multivalent supramolecular assemblies, highlighting how this shift overcomes key limitations while expanding functionality. Recent advances in conjugation chemistry, scaffold engineering, and protein design have enabled sophisticated affibody-based architectures with enhanced pharmacokinetic profiles and multivalent binding capabilities, thereby improving their utility in targeted drug delivery, molecular imaging, and theranostics.

## 1. Introduction

Affibody molecules, engineered derivatives of the Z-domain from *Staphylococcus aureus* protein A, offer promising alternatives to conventional antibodies. Their small size (~6.5 kDa), high stability, and engineerable binding interfaces provide key advantages, including efficient tissue penetration, thermostability, and tunable affinity/specificity for diverse targets such as proteins, peptides, and small molecules [1,2]. These properties position affibodies as valuable tools in targeted drug delivery, molecular imaging, diagnostics, biosensor development, and proteomics research.

Despite these strengths, monomeric affibody constructs have inherent limitations, including rapid renal clearance and short in vivo half-life [2,3]. To address these, researchers have shifted toward multivalent supramolecular architectures. Constructed via strategic non-covalent interactions, these architectures mitigate monomeric shortcomings while enabling new functions, such as enhanced target avidity, improved pharmacokinetics, controlled therapeutic release, and amplified diagnostic signals [4,5,6]. Moreover, they facilitate integration of multiple functionalities, supporting advanced therapeutic delivery systems and biomaterials.

This review focuses on recent advances in affibody-based supramolecular architectures, with emphasis on their applications in diagnostics, targeted therapeutics, and biomaterial development.

## 2. Brief History and Development of Affibodies

Affibody technology originated in the early 1990s at the Royal Institute of Technology (KTH) in Stockholm, Sweden, to address limitations of conventional antibodies [7]. While antibodies provide high specificity, their large size, poor tissue penetration, and complex production hinder many applications. The key innovation involved engineering the Z-domain of Staphylococcal Protein A into small proteins that retain high specificity and affinity while offering superior properties [1,2,3,7,8].

**Figure 1:**
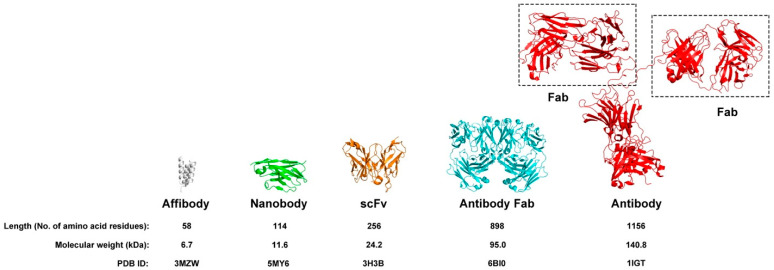
Crystal structures of human epidermal growth factor receptor 2 (HER2)-binding proteins and IgG2a monoclonal, scaled to their actual molecular sizes: affibody (PDB ID: 3MZW; gray), Nanobody (PDB ID: 5MY6; green), single-chain Fv (scFv) fragment of an anti-HER2 antibody (PDB ID: 3H3B; orange), Trastuzumab Fab (PDB ID: 6BI0; cyan), and IgG2a monoclonal antibody (PDB ID: 1IGT; red).

At approximately 6.5 kDa, affibodies are roughly one-twenty-fifth the size of conventional antibodies (Figure 1), conferring advantages including improved tissue penetration and simplified production through recombinant DNA technology [7]. The pivotal development involved modifying the Z-domain to introduce variability at specific binding regions, enabling creation of diverse affibody libraries capable of recognizing various target proteins with high specificity [1,2,3,7,8].

The evolution of affibody technology accelerated through advances in protein engineering and molecular biology. Key developments included implementation of phage display and yeast surface display techniques, enabling generation of diverse affibody libraries with picomolar binding affinities for critical targets including human epidermal growth factor receptor 2 (HER2) and epidermal growth factor receptor (EGFR) which are important markers in cancer diagnostics and therapeutics [7,8,9,10,11,12,13,14].

Affibodies rapidly gained traction across multiple scientific and clinical domains. In targeted therapeutics, they facilitate delivery of cytotoxic agents directly to cancer cells expressing specific biomarkers, minimizing off-target effects while enhancing treatment efficacy [8,9,10,15,16,17]. In molecular imaging, affibodies labeled with radionuclides have proven valuable for positron emission tomography (PET) and single-photon emission computed tomography (SPECT), enabling precise visualization of disease-associated targets in vivo [17,18,19,20,21]. Additionally, these molecules contribute significantly to biosensor development, where their specificity and stability enhance detection sensitivity and reliability [8,9,10,17].

The continuous innovation in protein engineering, molecular biology, and biotechnological applications has driven substantial development of affibody technology since its inception. The unique properties of affibodies (i.e., small size, high stability, and exceptional binding specificity), combined with scalable production methods and versatile application potential, have established them as promising platforms for diagnostic and therapeutic applications. Ongoing research continues expanding the capabilities and applications of these molecules, underscoring their importance in molecular medicine and biotechnology.

## 3. Molecular Structure and Scaffold Description

Affibody molecules represent a distinctive class of engineered proteins derived from the Z-domain of Staphylococcal Protein A [7]. They are characterized by their remarkably compact structure, comprising 58 amino acids with a molecular mass of approximately 6.5 kDa [7]. 

**Figure 2:**
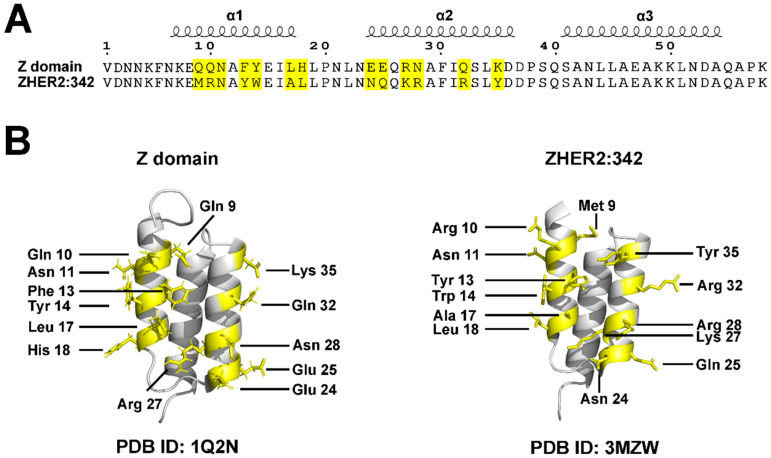
Sequence alignment and crystal structures of affibodies. (**A**) Sequence alignment of the Z domain and ZHER2:342 affibody, with identical residues shaded in black and the 13 mutated residues highlighted in yellow. (**B**) Crystal structures of the Z domain (PDB ID: 1Q2N) and ZHER2:342 affibody (PDB ID: 3MZW), with the 13 mutated residues highlighted in yellow to illustrate structural differences.

The molecular architecture features a characteristic three-helix bundle configuration (α1, α2, and α3) stabilized by a central hydrophobic core of strategically positioned nonpolar amino acids (Figure 2) [22,23,24]. Notably, this stable structure does not require disulfide bonds, distinguishing affibodies from many other protein scaffolds and contributing to their robust stability under reducing conditions.

A defining structural feature of affibody molecules is their adaptable terminal regions. Both N- and C-termini serve as versatile attachment points for various functional groups, enabling precise molecular engineering while preserving the essential three-helix bundle conformation [13,24,25,26]. This structural characteristic renders affibodies highly adaptable for diverse applications requiring site-specific modifications.

### 3.1. Binding Properties and Affinity

**Figure 3:**
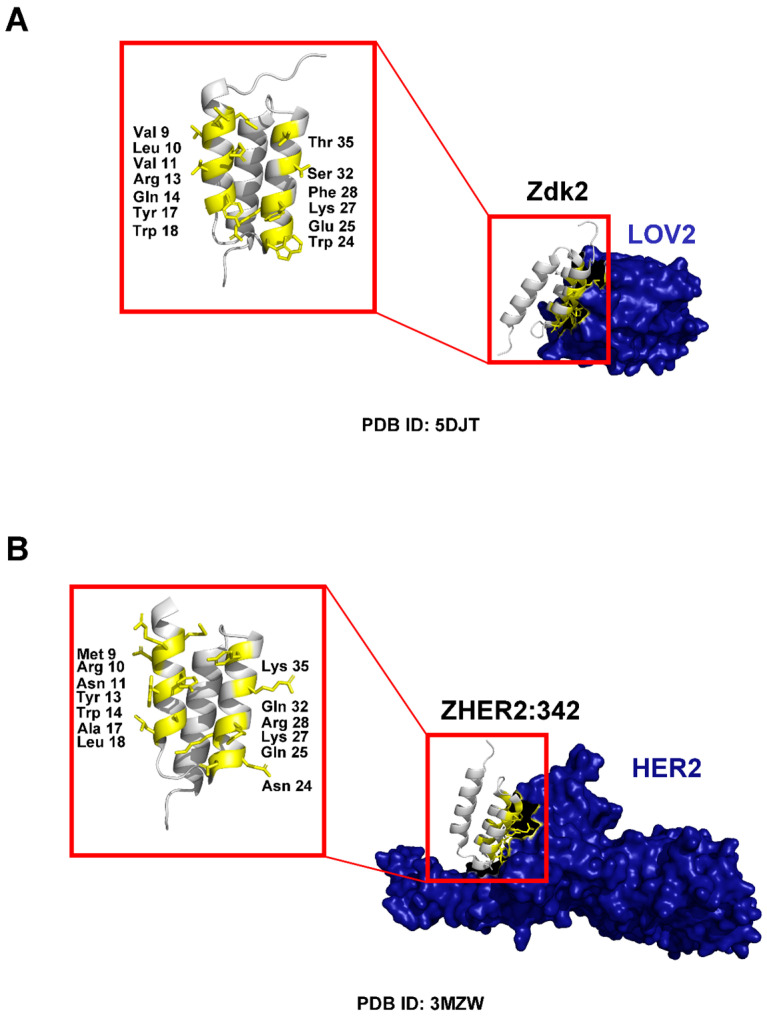
Crystal structures of affibody-receptor complexes. (**A**) The Zdk2-LOV2 complex (PDB ID: 5DJT), with the 13 mutated residues highlighted in yellow on the Zdk2 affibody. (**B**) The ZHER2:342-HER2 complex (PDB ID: 3MZW), with the 13 mutated residues highlighted in yellow on the ZHER2:342 affibody.

Affibody molecules achieve high specificity and affinity through engineered surface binding interfaces. Their binding properties primarily derive from modifications to 13 specific amino acid residues located on the first two helices (α1 and α2) of the three-helix bundle (Figure 2 and Figure 3) [22,23,24]. These residues constitute the primary binding interface and serve as key interaction points with target proteins. Through targeted mutagenesis of these 13 positions, either through random mutations or rational design, researchers generate diverse affibody libraries exhibiting wide-ranging binding specificities and affinities [1,2,8,11,12,13,14,22,23,24,27]. This flexibility enables affibodies to recognize and bind a broad spectrum of targets, including receptors, enzymes, and clinically relevant proteins, with remarkable precision [2,3,8,17,28,29]. Recent studies have demonstrated that next-generation affibody libraries with optimized randomization strategies can achieve binding affinities in the picomolar to low nanomolar range, rivaling those of monoclonal antibodies while maintaining the advantageous properties of small protein scaffolds [29]. For example, the well-established HER2-targeting affibodies ZHER2:342 (KD~22 pM) and ABY-025 (KD~76 pM) demonstrate comparable binding strength to the HER2-targeting antibody Trastuzumab (Herceptin, KD~100 pM), highlighting affibodies’ potential for high-affinity applications with reduced molecular weight [9,27,30].

### 3.2. Stability

Affibody molecules exhibit exceptional structural resilience and stability, maintained even when amino acid substitutions are introduced at the thirteen binding residues. This robustness stems from the inherent stability of the Z-domain-derived scaffold, featuring a well-conserved folding pattern and hydrophobic core composed of nonpolar amino acid residues [22,23,24,27]. This architecture creates a stable framework that accommodates significant sequence variability without compromising the molecule’s overall conformation. Consequently, affibody molecules retain their functional and structural integrity despite extensive engineering, ensuring reliable performance across diverse applications [24,27]. This feature makes affibodies an ideal scaffold for generating combinatorial libraries and selecting variants with high specificity for diverse targets.

Affibodies demonstrate remarkable stability under challenging environmental conditions, including broad pH tolerance (pH 2–11), resistance to reducing conditions (due to the absence of disulfide bonds), compatibility with organic solvents including dimethyl sulfoxide (DMSO) and ethanol, high thermal stability (up to 90 °C), and resistance to proteolytic degradation [2,29]. These properties make them suitable for a wide range of therapeutic and diagnostic applications requiring stability under physiological and non-physiological conditions.

### 3.3. Half-Life

The small size of affibody molecules promotes rapid renal clearance, resulting in short circulatory half-life [1,2,3,17]. While this characteristic proves advantageous for diagnostic applications requiring swift elimination from non-target tissues, it presents limitations for therapeutic applications where extended circulation time is desirable [1,2,3,17]. To address this constraint, researchers have developed modification strategies, including polyethylene glycol (PEG) conjugation and incorporation of albumin-binding domains, to extend the in vivo half-life of affibodies while preserving their binding functionality [3,31,32]. Recent work by Zhang et al. (2024) has demonstrated that albumin binding domain (ABD)-fusion leads to significantly higher tumor uptake compared to other half-life extension strategies like PASylation and XTENylation, while maintaining favorable biodistribution profiles for therapeutic applications [32]. This represents an important advancement in addressing one of the key limitations of affibody molecules for therapeutic use.

## 4. Basic Affibody Monomeric Constructs

Basic affibody monomeric constructs fall into two primary categories: monopartite and multipartite designs. Monopartite affibody constructs consist solely of the engineered affibody domain, emphasizing simplicity in production and characterization. These designs find application in receptor inhibition, biosensor development, and neutralizing inhibition strategies [1,2,3,8,17,29].

Multipartite affibody designs expand upon the basic structure by incorporating a linker, either functional or non-functional, and at least one additional functional unit. These units may include peptides, proteins, organic molecules, or detection probes. This modular architecture enables diverse applications, including bispecific targeting, drug conjugation with optimized release kinetics, enzyme attachment for localized therapeutic activity, and imaging applications using chelators such as 1,4,7,10-tetraazacyclododecane-1,4,7,10-tetraacetic acid (DOTA) for PET/SPECT imaging [1,2,3,8,17,20,21,29]. Additionally, these constructs can be integrated into advanced protein degradation systems, including Proteolysis Targeting Chimeras (PROTAC) [33,34].

Functionalization of affibodies can be achieved through either chemical conjugation, where moieties are attached via chemical linkers, or genetic incorporation, where functional groups are introduced during protein synthesis [1,2,3]. Each approach offers distinct advantages depending on the specific application requirements.

The advantages of monomeric affibody constructs include their small size, facilitating rapid tissue penetration; high affinity and specificity for targeted binding; straightforward production methods; and exceptional stability [1,2,3,17,29]. However, their short serum half-life due to rapid renal clearance remains a significant limitation, particularly problematic for therapeutic applications requiring prolonged circulation [1,2,3]. Strategies such as PEGylation, PASylation, XTENylation, and incorporation of albumin-binding domains are employed to extend their in vivo half-life while maintaining functional activity [3,31,32].

Clinical studies provide empirical evidence of these strengths and limitations in practice. For instance, monomeric affibody-based imaging probes, such as ^68^Ga-NOTA-MAL-Cys-MZHER2:342 and ^99m^Tc-ZHER2:41071, have shown promising advances in visualizing HER2 expression in breast cancer, achieving high sensitivity and specificity for detecting HER2-positive lesions shortly after injection [35,36,37]. These probes exhibit rapid blood clearance (distribution half-life~4–5 min, elimination~3–4 h), and predominant kidney accumulation within 1–2 h, resulting in high renal doses and restricted utility beyond short-term diagnostic imaging [35,36,37]. While these properties enable high-contrast PET/SPECT imaging within 1–2 h post-injection, they underscore challenges for therapeutic uses, such as achieving prolonged tumor exposure or minimizing off-target effects. This highlights the potential of supramolecular architectures, as discussed in subsequent sections, to address these issues by enhancing circulation and biodistribution.

## 5. Multivalent Assembly of Affibody Molecules

Despite their small size and high binding affinity, affibody molecules face a critical in vivo limitation: short circulatory half-life due to rapid renal clearance. Supramolecular assembly strategies address this by enhancing pharmacokinetic profiles and enabling versatile, multivalent systems for therapeutic and diagnostic applications [4,5,6,38,39,40,41].

For instance, Xia et al. (2022) developed ~153 nm nanomicelles by conjugating HER2-targeting affibody ZHER2:342-Cys to monomethyl-auristatin E (MMAE) [42]. These nanomicelles demonstrated prolonged blood circulation (fluorescence~0.86 × 10^4^ a.u. at 8 h) compared to monomeric counterparts (~0.4 × 10^4^ a.u.), alongside reduced renal clearance, enhanced Her2-positive SKOV-3 tumor accumulation (up to 8 h), and near-complete tumor eradication in HER2-positive ovary (SKOV-3) and breast (BT474) models [42]. Similarly, Xia et al. (2024) created another ~72.5 nm nanomicelles from a HER2-Affibody-epothilone B conjugate, exhibiting slower blood clearance (~5.6 µg/mL at 12 h) than free affibody (~0.4 µg/mL), greater tumor retention, and exceptional tumor inhibition at 10 mg/kg [43]. For EGFR targeting, Yang et al. (2025) created ~90 nm nanomicelles by linking ZEGFR:1907 Affibody with MMAE, achieving an extended half-life (~2.14 h) over monomers (<0.5 h), 4.2-fold greater tumor accumulation in EGFR-positive (A431) models at 8 h, and 97.3% tumor inhibition rate [44]. Collectively, these supramolecular architectures overcome monomeric affibodies’ limitations by enlarging particle size to slow down renal clearance, extend blood circulation, and improve tumor-specific biodistribution, thereby facilitating sustained drug delivery and superior therapeutic efficacy in preclinical settings.

### 5.1. Supramolecular Incorporation Strategies

**Figure 4:**
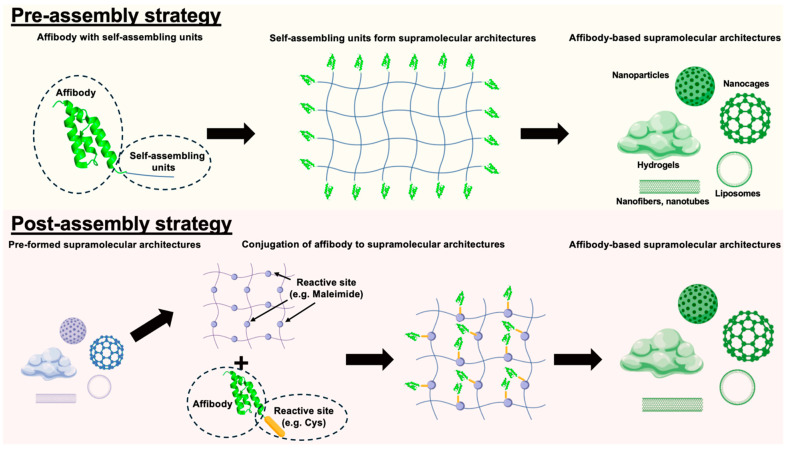
General illustration of the pre-assembly and post-assembly strategies for affibody incorporation into supramolecular scaffolds. Green represents supramolecular architectures with affibody functionalization. Blue represents supramolecular architectures without affibody functionalization.

Supramolecular chemistry leverages non-covalent interactions, including hydrogen bonding, π–π stacking, and van der Waals forces, to construct highly organized, functional biomolecular architectures [45]. Researchers harness these to integrate affibody molecules, combining their target-binding strengths with the adaptability, stability, and multivalency of supramolecular frameworks [38]. To the best of our knowledge, pre-assembly and post-assembly strategies are the two primary approaches that guide affibody integration into supramolecular scaffolds (Figure 4).

#### 5.1.1. Pre-Assembly Incorporation Strategies

In pre-assembly strategies, affibody molecules are modified through genetic fusion or post-expression chemical and biological methods to incorporate self-assembling units (e.g., peptides, polymers, or DNA scaffolds) before scaffold formation. The key advantage lies in enabling precise, site-specific integration through the introduction of reactive groups, such as azides, alkynes, or thiol, without interfering with the function of these self-assembling units. For instance, site-directed mutagenesis can introduce cysteine residues for thiol-maleimide reactions, while genetic code expansion enables the incorporation of non-natural amino acids for bioorthogonal click chemistry. High-affinity non-covalent partners, like biotin–streptavidin, serve as effective alternatives to these covalent methods. As the modified affibodies self-organize into micelles, vesicles, fibers, or hydrogels, they achieve optimized positioning for target binding.

This method provides exceptional spatial precision, enabling researchers to adjust affibody orientation and distribution. Binding domains thus remain accessible and functional, which is essential for targeted therapeutics and high-resolution imaging [42,46,47,48]. Moreover, uniform modifications ensure consistent functionality across batches, improving experimental reproducibility. Pre-assembly also supports multivalent configurations, where multiple affibody units synergize to boost target recognition via avidity effects [3,5].

However, this approach requires rigorous chemistry to preserve affibody folding and binding functionality. Scalability challenges may arise when complex purification steps prove difficult to replicate at industrial scale. Additionally, affibodies must be protected from destabilizing assembly conditions (such as heat, pH shifts, or organic solvents) that could impair molecular recognition.

##### Examples of Pre-Assembly Incorporation in Affibody-Based Supramolecular Architectures

Pre-assembly incorporation involves integrating affibody molecules into supramolecular structures during the initial formation process, allowing for precise control over architecture, stoichiometry, and functionality. This strategy contrasts with post-assembly methods by embedding the targeting moiety within the scaffold, often enhancing stability, multivalency, and pharmacokinetic properties. Below, we highlight recent examples that demonstrate the versatility of pre-assembly in therapeutic and diagnostic applications (Table 1).

One exemplary approach is to integrate affibodies into virus-like particles (VLPs) for targeted delivery, leveraging genetic fusion for seamless incorporation during assembly. In a study by Nishimura et al. (2013), HER2-specific affibodies were genetically fused to hepatitis B core (HBc) proteins. During recombinant expression in *E. coli*, these fusion proteins self-assembled into hollow HBc particles. This pre-assembly conferred high specificity for HER2-overexpressing breast cancer cells (e.g., SK-BR-3), with enhanced cellular uptake [49].

Building on well-known amphiphile self-assembly mechanisms, affibody-cytotoxin conjugates have been pre-incorporated into nanoagents for targeted cancer therapy, capitalizing on the conjugates’ inherent ability to form nanostructures. For instance, Yang et al. (2025) described EGFR-targeted affibodies conjugated to monomethyl auristatin E (MMAE) through thiol-maleimide chemistry [44]. Due to the amphiphilic nature, the affibody-MMAE conjugates self-assembled into nanoagents. The resulting nanoagents exhibited rapid internalization, potent cytotoxicity in EGFR-positive cancer cells, as well as strong tumor targeting and antitumor effects in vivo. This pre-assembly method improved tumor penetration and reduced systemic toxicity compared to free conjugates, highlighting its promise for precise cancer targeted therapy [44]. Similarly, Xia et al. (2024) conjugated HER2-targeted affibodies to epothilone B via thiol-maleimide chemistry [43]. The amphiphilic affibody-drug conjugates self-assembled into core–shell nanoagents for enhanced release and efficacy in HER2-positive breast and ovarian tumors [43]. Recent advances have also leveraged self-assembling affibody-PROTAC conjugates for targeted delivery. For PROTAC-based systems, Gao et al. (2024) and Li et al. (2024) described amphiphilic conjugates where EGFR- or HER2-targeted affibodies (ZEGFR:1907 or ZHER2:342) were linked to PROTACs (MS28 or MZ1) via bioresponsive linkers [33,34]. These amphiphilic affibody-PROTAC conjugates self-assembled into nanoagents (e.g., ADCN or APCN) in aqueous solutions. This enhanced tumor accumulation, controlled release, and degradation of targets like cyclin D1 or BRD4 in EGFR- or HER2-positive models, achieving high biosafety and antitumor efficacy [33,34].

Apart from amphiphile self-assembly systems, thermoresponsive self-assembly systems have also been designed to integrate affibody molecules into supramolecular structures. For example, Li et al. (2024) fused HER2-targeted affibodies (ZHER2:342) to elastin-like polypeptides (ELP) and conjugated them to MMAE, forming thermoresponsive nanomicelles during self-assembly above the transition temperature [50]. This pre-assembly improved HER2-mediated endocytosis and tumor penetration in ovarian cancer xenografts [50].

Peptide-driven self-assembly offers another avenue to generate affibody supramolecular architectures for diagnostic applications. In Liu et al. (2022), affibodies targeting alpha-fetoprotein (AFP), a biomarker for liver cancer, were pre-assembled with β-sheet-forming peptides to create bioactive nanofibril aggregates [51]. The affibodies were genetically fused to self-assembling peptides prior to aggregation, resulting in structures with enhanced stability and improved immunoassay signal amplification activity. This system achieved limits of detection as low as 9.7 ng mL^−1^ AFP, far surpassing traditional ELISA methods, and underscores the role of affibody-based supramolecular architectures in biosensing platforms [51].

DNA-based nanostructures further expand pre-assembly possibilities for synergistic therapies using affibody supramolecular architectures. Zhang et al. (2022) reported HER2-targeted affibodies modified onto G-quadruplex DNA micellar prodrugs, which self-assembled into G-quadruplex DNA micelles loaded with polymeric 5-fluorodeoxyuridine (5-FdU) and curcumin during micelle formation [52]. The pre-incorporated affibodies facilitated receptor-mediated endocytosis in HER2-positive gastric cancer cells, enabling dual-drug release that synergistically inhibited tumor growth in vivo [52]. Similarly, Zhang et al. (2020) utilized affibody-conjugated RALA amphipathic peptides to form nanoparticles encapsulating oligomeric 5-FdU [53]. Pre-assembly ensured uniform distribution of the targeting ligands, leading to superior efficacy in HER2-overexpressing gastric cancer models [53]. These innovative applications demonstrate the broad utility of affibody-based supramolecular architectures in enhancing drug delivery systems and enabling controlled release of multiple therapeutic agents.

Finally, hydrogel-based systems represent a culminative evolution of these pre-assembly strategies, incorporating affibodies into biocompatible matrices for long-term therapeutic delivery in regenerative contexts. Teal et al. (2022) incorporated affibody-modified methylcellulose into biopolymer-based hydrogels to achieve sustained and independent release of therapeutic proteins over seven days [54]. This methodology demonstrated significant applications in tissue regeneration and retinal degenerative disease treatment, offering versatile strategies for simultaneous delivery of multiple therapeutics [54].

**Table 1:** Summary of Pre-assembly Incorporation Examples in Affibody-based Supramolecular Architectures.

Affibody Targets	Supramolecular Architectures	Self-Assembling Units	Payloads	Applications	Citation
HER2	Virus-like particles	Hepatitis B core (HBc) proteins	Fluorescent probe (e.g., Alexa Fluor 488)	Targeted delivery	Nishimura et al. (2013) [49]
EGFR	Affibody-drug conjugate-based nanoagent (nanomicelles)	MMAE (amphiphile)	Anti-cancer drug (e.g., MMAE) & fluorescent probe (e.g., Cy5.5)	Targeted delivery	Yang et al. (2025) [44]
HER2	Affibody-drug conjugate-based nanoagent (nanomicelles)	Epothilone B (amphiphile)	Anti-cancer drug (e.g., Epothilone B) & fluorescent probe (e.g., Cy5.5)	Targeted delivery	Xia et al. (2024) [43]
EGFR	Affibody-PROTAC conjugate-based nanoagent (nanomicelles)	PROTAC MS28 (amphiphile)	PROTAC (e.g., MS28) & fluorescent probe (e.g., Cy5.5)	Targeted delivery	Gao et al. (2024) [33]
HER2	Affibody-PROTAC conjugate-based nanoagent (nanomicelles)	PROTAC MZ1 (amphiphile)	PROTAC (e.g., MZ1) & fluorescent probe (e.g., Cy5.5)	Targeted delivery	Li et al. (2024) [34]
HER2	Nanomicelles	Elastin-like peptide (ELP)-MMAE	Anti-cancer drug (e.g., MMAE) & fluorescent probe (e.g., Cy5)	Targeted delivery	Li et al. (2024) [50]
AFP	Affibody aggregates	Self-assembly peptides	-	AFP detection	Liu et al. (2022) [51]
HER2	Nanomicelles	G-quadruplex DNA	Anti-cancer drug (e.g., 5-FdUR and curcumin) & fluorescent probe (e.g., FAM)	Targeted delivery	Zhang et al. (2022) [52]
HER2	Nanoparticles	RALA amphipathic peptides	Anti-cancer drug (e.g., FUdR_15_) & fluorescent probe (e.g., FAM)	Targeted delivery	Zhang et al. (2020) [53]
IGF-1 or PEDF	Hydrogel	Methylcellulose	IGF-1 or PEDF	Controlled release of therapeutic proteins	Teal et al. (2022) [54]

#### 5.1.2. Post-Assembly Incorporation Strategies

Post-assembly strategies involve attaching affibodies to preformed supramolecular scaffolds, offering enhanced design flexibility. The process begins with synthesizing and characterizing nanostructures (such as liposomes, polymeric micelles, protein cages, or inorganic nanoparticles), focusing on key parameters like size distribution, surface charge, and stability [55].

Affibodies are attached to these preformed scaffolds using covalent bonds or usually high-affinity non-covalent interactions functionally equivalent to covalent bonds, enabling sequential functionalization. Covalent attachment methods, including thiol–maleimide chemistry, click reactions, 1-ethyl-3-(3-(dimethylamino)propyl)carbodiimide/N-hydroxysuccinimide (EDC/NHS) coupling, SpyTag/SpyCatcher systems, and HaloTag technology, provide robust long-term stability through irreversible linkages. Alternatively, non-covalent approaches such as biotin–streptavidin pairs, electrostatic forces, hydrophobic interactions, or molecular recognition motifs can also be used to functionalize these preformed scaffolds.

A major strength of post-assembly is its modularity, allowing sequential addition of distinct affibodies to create multifunctional systems for dual-targeting or combined imaging and therapy. This adaptability supports customization for specific research or clinical needs while preserving the scaffold’s structural integrity and functional properties throughout functionalization [56,57]. For instance, functionalizing existing nanoparticle systems such as gold nanoparticles and quantum dots can enhance imaging signal intensity or improve drug delivery precision [48,58,59,60,61]. Similarly, attaching affibodies to protein cages, including virus-like particles, can enhance pharmacokinetic control and therapeutic efficacy [17,62,63].

Despite this versatility, post-assembly offers less precise control over affibody spatial distribution and orientation compared to pre-assembly, which may lead to heterogeneous binding. Achieving uniform functionalization across scaffolds can be challenging, underscoring the need for optimized conjugation protocols and rigorous characterization to ensure reliable performance.

##### Examples of Post-Assembly Incorporation in Affibody-Based Supramolecular Architectures

Post-assembly incorporation refers to the attachment of affibody molecules to pre-formed supramolecular structures, such as nanoparticles, beads, or bubbles, after their initial synthesis or assembly. This approach allows for modular functionalization, enabling precise targeting while preserving the core properties of the scaffold, including stability, imaging capabilities, and biocompatibility. In contrast to pre-assembly methods, post-assembly strategies often simplify production and facilitate the addition of multiple ligands for multimodal applications. Below, we highlight recent examples that showcase the versatility of post-assembly in diagnostic, imaging, and therapeutic contexts (Table 2).

Nanoparticle-based systems exemplify post-assembly’s utility in multimodal imaging, where affibodies are conjugated to pre-formed cores to boost targeting specificity and signal amplification. For instance, Jokerst et al. (2011) functionalized gold–silica nanoparticles (SERS NPs) with EGFR-specific affibodies after silica shell formation, linking them via a short PEG-maleimide cross-linker to thiol groups on the surface [58]. This enabled Raman molecular imaging of EGFR-positive A431 tumors, achieving a signal nearly 35-fold higher in positive tumors compared to EGFR-negative ones [58]. Similarly, Yang et al. (2013) modified gold–iron oxide heteronanostructures with EGFR-specific affibodies post-assembly, incorporating radiolabels for PET, optical, and magnetic resonance imaging (MRI) of EGFR-expressing tumors, resulting in high tumor uptake and multimodal contrast via maleimide chemistry [48]. Gao et al. (2012) also explored affibody-conjugated quantum dots and iron oxide nanoparticles for imaging HER2-expressing cells and tumors [38]. Incorporating anti-HER2 affibodies onto these supramolecular architectures using maleimide chemistry resulted in high specificity and affinity for HER2-positive cancer cells, making them effective for both fluorescence and MRI-based diagnostic applications. This dual-modality approach enhances tumor detection precision, providing comprehensive diagnostic capabilities [38]. These imaging-focused examples illustrate how post-assembly leverages plasmonic or magnetic properties for sensitive detection, paving the way for sensitive cancer diagnosis.

Building on these imaging foundations, nanoparticle systems have evolved to incorporate post-assembly affibodies for targeted therapy, combining diagnostic capabilities with potent antitumor effects. Extending to photothermal applications, Shipunova et al. (2022) conjugated HER2-specific affibodies (ZHER2:342) to pre-synthesized silver nanoparticles via a PEG linker, enabling targeted photothermal therapy (PTT) in HER2-overexpressing tumors [64]. The post-assembled Ag-PEG-HER2 NPs demonstrated efficient ROS generation and heating under light irradiation, leading to complete tumor regression in xenograft models [64]. Complementing this, Shipunova et al. (2021) decorated pre-formed poly(lactic-co-glycolic acid) (PLGA) nanoparticles with anti-HER2 affibodies post-assembly for targeted delivery of photosensitizers, inducing photoactivated cell death upon light irradiation in HER2-positive breast cancer cells with high specificity and minimal off-target effects [65].

Bubble-based architectures offer innovative post-assembly strategies for ultrasound imaging and combined photodynamic treatments. Yang et al. (2015) conjugated biotinylated anti-HER2 affibodies to pre-formed phospholipid nanobubbles via streptavidin-biotin bridging, producing targeted ultrasound contrast agents for HER2-overexpressing breast tumors, with peak intensities of 104.5 dB and high tumor selectivity [66]. This innovation improved molecular ultrasound imaging specificity and offers potential for real-time monitoring of therapeutic responses using ultrasound imaging [66]. Similarly, enhancing this targeted nanobubble concept with combined photodynamic therapy (PDT), Cai et al. (2023) conjugated HER2-specific affibodies to pre-formed nanobubbles loaded with near-infrared photothermal probe IR783 and photosensitizer 2-[1-hexyloxyethyl]-2-devinyl pyropheophorbide-a (HPPH) through a biotin–streptavidin system to form nanobubble-IR783-HPPH-affibody (NIHA) complexes, enabling laser-activated PDT for HER2-positive breast cancer, with significant increases in ROS production and tumor cell death in vitro and in vivo [67]. The combination of PDT with targeted delivery significantly reduced tumor viability, highlighting the potential of affibody-functionalized nanoparticles in non-invasive cancer treatment.

Protein nanocage-based systems further advance post-assembly by integrating affibodies into porous protein-based scaffolds for enhanced delivery and synergistic effects. In a study by Kim et al. (2021), Gd(III)-DOTA protein cage nanoparticles were post-functionalized with multiple HER2 or EGFR-specific affibodies using a SpyTag/SpyCatcher system, enabling target-switchable T1 contrast in high-field MRI with selective binding to HER2 or EGFR-overexpressing cells for highly specific and sensitive cancer diagnosis [68]. Similarly, Eom et al. (2024) engineered porous SpyCatcher-mi3 protein cage nanoparticles as modular delivery platforms, post-displaying affibodies for ligand-targeted cargo release in therapeutic applications, enabling precise targeting and delivery of chemotherapeutic agents to EGFR-overexpressing cancer cells [69]. Extending to protein nanocage nanoparticle systems for intracellular drug delivery, Jun et al. (2022) displayed both tumor neutralizing factor-related apoptosis-inducing ligand (TRAIL) and EGFR-specific affibodies on pre-formed lumazine synthase protein cage nanoparticles post-assembly, synergistically inducing apoptosis and suppressing tumor growth in EGFR-overexpressing models through combined receptor targeting and death ligand activation [62].

Apart from protein nanocage systems, metal–organic framework (MOF) systems are another promising nanoparticle-based vehicle for drug delivery. Oh et al. (2023) coated pre-formed Zr-based MOF nanoparticles (PCN-224) with GST-fused HER2- or EGFR-specific affibodies to form a protein–MOF hybrid system, creating a protective protein corona shield that minimized serum protein adsorption and enabled targeted delivery of camptothecin for synergistic PDT and chemotherapy in breast cancer models [70].

DNA-based nanostructures further expand post-assembly possibilities for targeted drug delivery using affibody-incorporated supramolecular architectures. Yu et al. (2023) developed CytoDirect, a disulfide-modified DNA origami nanodevice functionalized with HER2 affibodies for efficient cytosolic delivery of therapeutic payloads [71]. By integrating affibodies post-assembly through an oligonucleotide handle, CytoDirect achieved high specificity and enhanced cellular uptake in HER2-positive cancer cells, facilitating precise delivery of therapeutic oligonucleotides and doxorubicin [71].

Affibody-incorporated supramolecular architectures also contribute to the area of controlled drug release. Dorogin et al. (2023) investigated controlled delivery of bone morphogenetic protein-2 (BMP-2) for bone regeneration by conjugating BMP-2-specific affibodies to polyethylene glycol–maleimide hydrogels [72]. This system enabled precise modulation of BMP-2 release kinetics, enhancing osteogenic stimulation while minimizing adverse effects and offering promising strategies for improving bone healing outcomes [72]. In addition to covalent linkages, Liang et al. (2018) leveraged the concept of self-assembling peptides forming co-assembled nanofibers with proteins during the self-assembly process [40]. They developed a novel therapeutic approach for targeting HER2-positive tumors by co-assembling drug-peptide amphiphiles with anti-HER2 affibodies, resulting in supramolecular nanofibers embedded with the affibodies. The resulting nanofibers demonstrated significant suppression of HER2^+^ tumor growth in vivo, showing improved accumulation and retention in tumors [40].

**Table 2:** Summary of Post-assembly Incorporation Examples in Affibody-based Supramolecular Architectures.

Affibody Targets	Supramolecular Architecture	Affibody Conjugation Method	Payloads	Applications	Citation
EGFR	Gold–silica nanoparticles	Thiol/maleimide	-	Targeted delivery for Raman imaging	Jokerst et al. (2011) [58]
EGFR	Gold–iron oxide hetero-nanostructures	Thiol/maleimide	-	Targeted delivery for PET, Optical and MR Imaging	Yang et al. (2013) [48]
HER2	Quantum dots and iron oxide nanoparticles	Thiol/maleimide	-	Targeted delivery for optical and MR Imaging	Gao et al. (2011) [38]
HER2	Silver nanoparticles	NHS/EDC	-	Targeted delivery for photothermal therapy	Shipunova et al. (2022) [64]
HER2	PLGA nanoparticles	NHS/EDC	Photosensitizer (e.g., Rose Bengal)	Targeted delivery for photosensitizer	Shipunova et al. (2021) [65]
HER2	Phospholipid nanobubble	Biotin/streptavidin	-	Targeted delivery for ultrasound contrast agents	Yang et al. (2015) [66]
HER2	Phospholipid nanobubble	Biotin/streptavidin	Photothermal agents (e.g., IR783) & photosensitizer (e.g., HPPH)	Targeted delivery for ultrasound contrast agents, photothermal therapy & photosensitizer	Cai et al. (2023) [67]
HER2/ EGFR	Lumazine synthase protein nanoparticles	SpyTag/SpyCatcher	Contrast agents (e.g., Gd(III)-DOTA) & fluorescent probe (e.g., Alexa680)	Targeted delivery for optical and MR Imaging	Kim et al. (2021) [68]
EGFR	SpyCatcher-mi3 protein nanoparticles	SpyTag/SpyCatcher	Anti-cancer drug (e.g., aldoxorubicin) & fluorescent probe (e.g., Fluorescein, Alexa647)	Targeted delivery	Eom et al. (2024) [69]
EGFR	Lumazine synthase protein nanoparticles	SpyTag/SpyCatcher	TRAIL	Targeted anti-cancer therapeutic	Jun et al. (2022) [62]
HER2/EGFR	Zr_6_-based MOF nanoparticles (PCN-224)	Adsorption	Anti-cancer drug (e.g., Camptothecin)	Targeted delivery	Oh et al. (2023) [70]
HER2	DNA origami nanodevice	Thiol/maleimide/SMCC	Doxorubicin	Targeted delivery	Yu et al. (2023) [71]
BMP-2	Polyethylene glycol–maleimide hydrogels	Thiol/maleimide	BMP-2	Controlled release of therapeutic proteins	Dorogin et al. (2023) [72]
HER2	Nanofiber	Affibody embedded during co-assembly	-	Targeted delivery	Liang et al. (2018) [40]

## 6. Conclusions and Future Outlook

Affibody-based supramolecular architectures represent a pivotal advancement in protein engineering, transforming monomeric constructs into versatile multivalent systems with profound biomedical potential. By addressing inherent limitations like rapid renal clearance, these assemblies expand applications in therapeutics, diagnostics, biosensing, and biomaterials. Pre- and post-assembly strategies provide complementary tools for integration: pre-assembly excels in delivering precise spatial control, predictable multivalency, and uniform functionality, making it ideal for ordered arrays in complex scaffolds such as peptide assemblies or nucleic acid nanostructures. In contrast, post-assembly offers superior flexibility for sequential functionalization and scaffold preservation, enhancing existing platforms like nanoparticles and protein cages, though it may trade off some stability and precision.

Together, these approaches harness the robust binding specificity, stability, and adaptability of affibodies to create multifunctional platforms that outperform traditional methods. They enable precise targeting, extended pharmacokinetics, minimized off-target effects, and avidity-enhanced binding, driving innovations in cancer therapy, molecular imaging, and drug delivery. For instance, multivalent presentations facilitate efficient cellular uptake and therapeutic efficacy, while modular designs support hybrid systems for theranostics.

As the field progresses, ongoing refinements in conjugation chemistry, scaffold engineering, and protein design, coupled with computational modeling, advanced fabrication, and deeper biological insights, will yield increasingly tailored architectures. Researchers are exploring hybrid covalent–non-covalent methods to balance stability and reversibility, alongside strategies for controlling assembly homogeneity, reducing immunogenicity, and scaling production. These developments position affibody-based systems at the vanguard of personalized medicine and molecular diagnostics, promising adaptable solutions for diverse clinical challenges and accelerating transformative translations from bench to bedside.

## Data Availability

No new data were created or analyzed in this study. Data sharing is not applicable to this article.
